# Expansion of India’s national child healthcare programme, Rashtriya Bal Swasthya Karyakram (RBSK), for rare disease management : a health policy perspective

**DOI:** 10.1186/s13023-023-02761-y

**Published:** 2023-06-12

**Authors:** Pragya Chaube, Arun K. Singh, Mohua Chakraborty Choudhury

**Affiliations:** 1 Department of Science & Technology, Centre for Policy Research, Indian Institute of Sciences, Bengaluru, India; 2 Institute of Public Health, Bengaluru, India; 3 India Institute of Medical Sciences, Jodhpur, India; 4 Ministry of Health & Family Welfare, Rashtriya Bal Swasthya Karyakram (RBSK), Government of India, New Delhi, India

**Keywords:** Rare diseases, Public health, Child health, Healthcare system, Low and middle income countries (LMIC), Rashtriya Bal Swasthya Karyakram (RBSK)

## Abstract

Rare diseases (RD) are severe and debilitating conditions. They are one of the leading causes of childhood mortality globally. In India, RDs have not been considered in most healthcare programs which usually cater to more common diseases. We believe, that for efficient utilization of resources in a resource-constrained healthcare system, existing programs must integrate RD management strategies. In this study, we explore the utility, expandability, and limitation of one of the important national child healthcare programs, Rashtriya Bal Swasthya Karyakram (RBSK) which translates to National Child Healthcare Program. We found that RBSK has immense potential to cater to RDs through some of its unique features, such as comprehensive screening, wide target age group, and efficient utilization of resources. We provide recommendations that can help to strengthen the present program. This study will inspire other low-resource countries to identify and expand existing public healthcare programs for RD management. Moreover, RBSK can serve as a model program to integrate RD management globally.

**Supplementary Information:**

The online version contains supplementary material available at 10.1186/s13023-023-02761-y.

## Introduction

Rare diseases (RD) are a heterogeneous group of severe and debilitating diseases that occur infrequently in a population. Individually they might be rare but together they affect 6–8% of the global population [1]. In India, the actual burden of RDs is not known although it is estimated that there could be 72–96 million people living with RDs (PLwRDs) [2]. Significant proportion (50–75%) of RDs have their onset in childhood and birth defects and childhood disorders form a major subset [3, 4]. India does not have prevalence data for pediatric RDs but various child health indicator data [5] show that a huge population of children may be affected by some form of RDs. Studies estimate that nearly 1.7 million children are born with birth defects each year [5]. At 27.6 deaths per 1,000 live births, the infant mortality rate in India is significantly higher than other emerging economies like China (8.3) and developed nations like the US (5.4) [6]. This suggests that a considerable number of infants who may have an RD are not diagnosed in time and are lost.

RDs are a major economic burden on both the patients’ families and the country’s healthcare system. The patients’ families often have to bear the costs associated with the diagnostics odyssey of RDs, lifelong treatments, frequent incidents of hospitalization, and exorbitant drug prices [7]. Such individual experiences often translate to a large segment of the population when the entire RD community is taken into account, which exerts additional pressure on the country’s public healthcare system. A recent study found that the economic burden of RDs is ten times more than mass-market diseases [8]. Despite the cumulative burden of RDs on a nation, they are often ignored in the public health agenda due to the low frequency of individual diseases.

In India, only recently RDs have gained some recognition with the release of the revised draft of the National Policy for Rare Diseases (NPRD) in 2021. However, in a resource-constrained healthcare system, it may be difficult to have a completely separate route of management for RDs. Therefore, for efficient utilization of resources, it is necessary to integrate RD management strategies outlined in the NPRD into the existing public health programs. In a recent study, we mapped the potential of different programs under Reproductive, Maternal, Newborn, Child, and Adolescent Health (RMNCH + A) of National Health Mission initiatives to address RD management [9]. We found that Rashtriya Bal Suraksha Karyakram (RBSK) mapped to most of the RD management strategies identified in NPRD and thus has immense potential to cater to RDs. In this study, we take a deep dive into RBSK to understand the utility, expandability, and limitations of the program to extend it to RD management. This study makes suggestions for integrating RBSK into the RD management system. The authors present this position statement as representative of health policy researchers.

### Features of RBSK that have the potential for RD management

**Fig. 1:**
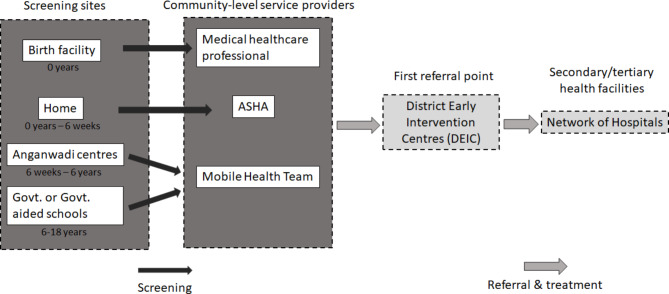
The schema for the RBSK mechanism. Image adapted from Deloitte & UNICEF, 2016 report [23]. Accessed February 28, 2022

RBSK was launched in 2013 under the National Health Mission with the vision to enable all children to achieve their full potential and provide comprehensive care to all the children in the community. RBSK, which roughly translates to a national child care program, employs a systemic approach of prevention, early identification, and management of thirty health conditions distributed under 4Ds: Defects at birth, Diseases, Deficiencies, and Developmental delays including Disabilities, to cater to children from birth to 18 years of age. RBSK utilizes community public healthcare resources for screening and relies on referrals and cross-referrals between secondary and tertiary healthcare units for efficient diagnosis and treatment management of these conditions. At the center of this mechanism are District Early Intervention Centers (DEIC) which are the first point of reference after screening and also play a key role in providing referrals, continuum of care, and record-keeping. The details of the functioning of RBSK are shown in Fig. 1 and in the subsequent section that discusses the features of RBSK which are useful for RD management. Further description of the functioning of RBSK is provided in Supplementary 1.

#### Comprehensive screening

Unlike the developed countries where prenatal and antenatal genetic screening is generally used, a more cost and resources effective screening methodology is required for Indian conditions. RBSK is the first of its kind comprehensive screening program in India that utilizes a cost and resource-effective screening methodology. It is comprehensive in three ways (a) ensures that a child is screened from head-to-toe (b) utilizes different modes of screening (c) targets a wide age group (0 to eighteen years).

- **Head to toe screening**.

RBSK recognizes that both screening and management require a holistic approach where different problems faced by a child are not screened by different specialists, rather by a single person who screens the child from head to toe. This procedure can provide full and timely insight into the health condition of a patient and can point to some early symptoms that may require early attention.

b) **Different modes of screening**.

RBSK encourages using different modes of screening to ensure the identification of a wide array of diseases. Starting from low-cost resource methods to more specialized and resource-intensive ones. Different modes of screening used for different types of birth defects are described in Table 1.

**Table 1:** Different modes of screening used for a different types of birth defects

Type Birth defect	Example	Mode of screening
Visible birth defect	Neural Tube defect, cleft lip, limb deformities, club foot	Visual screening. Detected through proper head to toe physical examination
Functional defects	Congenital deafness, congenital cataract, congenital glaucoma	Identified with the help of non-invasive instruments and clinical examination
Metabolic disorders	Congenital Hypothyroidism, congenital adrenal Hypoplasia, G6PD, Sickle cell disease	Through blood investigations. Newborn Blood spot screening
Chromosomal abnormalities and genetic disorders	Down syndrome	Through visible anthropometric measurements
Neurological disorders	Congenital Cerebral Palsy, IUGR (Intrauterine Growth Restriction) associated with birth defect	Through observing behavioral patterns and developmental delays

A visible birth defect is screened through visual screening by delivery point staff by head-to-toe examination. Guidelines for assessment and training programs are made available by RBSK. Functional birth defects are identified with the help of low-cost non-invasive instruments and clinical examination for which manpower can be easily trained, for example screening for vision defects through torch and opthalmoscope. To identify metabolic disordersnewborn blood investigations are done for a limited panel of diseases such as Congenital Hypothyroidism, congenital adrenal hypoplasia, glucose-6-phosphate dehydrogenase (G6PD) deficiency, sickle cell disease,. The RBSK resource mentions chromosomal abnormalities and genetic disorders and provides basic knowledge of these disorders. However, RBSK largely relies on non-invasive examination methods such as anthropometric measurements and developmental delays for the initial screening and identification of the patients. There is a lack of clarity on the diagnostic methods for exact diagnosis and on the details of how and at which point genetic testing will be facilitated. Nevertheless, RBSK documents lay out some instructions for genetic counselling.

c) **Wide target age group**.

RBSK envisions reaching a broad category of age groups. Each age category is assessed by different healthcare delivery staff for range of diseases/disorders and disabilities as described below in Table 2.

**Table 2:** Target age-group coverage of RBSK, details of screening and screening providers. Adapted from Operational Guidelines: RBSK [10]. Accessed February 28, 2022

Target age group	Who in this target age group will be screened?	What is screened?	Where and by whom is the screening done?
Birth to 6 weeks	Babies born at public health facilities and home -	The vital signs, anthropometric data, weight, dysmorphic features, fatigue, and other such visible phenotypes are assessed and recorded. This is followed by a tool-based and clinical examination. The cases that require urgent critical care for the neonate to survive, for example in case of respiratory distress, are referred for urgent treatment	At public health facilities by medical professionals
6 weeks to 6 years	Preschool children in rural areas and urban slums	More parameters are added to the physical head-to-toe screening. Children are also screened for deficiencies, developmental delays, and response to sensory stimuli, among others	MHT monitors child health twice a year at Anganwadi centers.
6 years to 18 years	School children enrolled in classes 1st and 12th in government and government-aided schools	Aside visible manifestation of 4Ds, signs of depression, behavioral assessment, performance in school, and other parameters are also assessed.	MHT monitors students’ health at government and government-aided schools once a year.

This holistic approach to assessing neonates and children is not only beneficial in improving child-health indicators and combating common child health issues such as infections and deficiencies but can also help in mapping RDs. RDs usually manifest as a multisystem disorder with a range of phenotypes that may include stunted growth, disability, issues with motor function, defective organogenesis, congenital defects, behavioral issues, etc [11]. These phenotypic markers are a crucial visual proxy to identify possible RDs. Monitoring children till the age of eighteen years provides an excellent opportunity to identify RDs with late-onset. Assessment based on behavior and developmental delays is an effective tool to tentatively identify major underlying issues. In India, children aged zero to eigtheen years comprise 40% of the population. If this population is routinely screened, as envisaged under RBSK, it can potentially prevent many childhood diseases, including RDs.

#### Lack of dependence on the parents

The community-based monitoring of child health is one of the major advantages of RBSK over the previous child screening programs implemented in the country. Previously, access to child healthcare was largely dependent on parents’ education, socio-economic background and their perception of health problems [12]. This was one of the key learnings from the Goa child health screening program (the first such program in the country launched in 2008). The dependence on parents’ willingness to participate had resulted in a lower rate of follow-up in the Goa program [13]. RBSK addresses this issue by making this process independent of parents and relying on community processes and the existing Accredited Social Health Activist (ASHA) network.

#### Efficient utilization of resources

RBSK advocates for screening by trained generalists and management by a specialist. A single screener focuses on screening different parts of the body of the child while its management is undertaken by other specialized professionals consisting of pediatricians, surgeons, and therapists [12]. RBSK advocates for the utilization of community public health workers and AYUSH doctors for screening. This reduces the burden on the professional medical staff and also reduces the waiting time for screening, diagnosis, and following downstream processes. The utilization of allied healthcare professionals in screening is particularly important in India as it suffers from one of the worst patients to doctor ratios [14]. Moreover, in India, home births account for 60.8% of all births, compared to health facilities or hospitals, which account for only 37.2% [15]. Therefore, engaging community public health workers is extremely important to reach out to larger population.

Rare diseases are resource-intensive as they require multiple specialists looking after multi-organ symptomatic presentation of diseases. Comprehensive screening through a generalist will lead to early identification of potential cases that need special attention and intervention.

However, RBSK recognizes that proper training and capacity building are crucial in ensuring that these professionals are equipped with the necessary knowledge and skills. RBSK has well-structured pathway for training and capacity building of professionals and provides comprehensive manual and job aids to guide the training process.

#### Record keeping

RBSK encourages the use of screening, tracking, and reporting software for data collection and analysis. It envisages capturing countrywide epidemiological data on the 4 Ds (i.e., Defects at birth, Diseases, Deficiencies, and Developmental delay including disabilities) to help in future planning of area-specific services or provide the fundamentals for further research. A major hindrance to a comprehensive RD management strategy is the lack of epidemiological data in the country [2]. RBSK has the potential to fill this knowledge gap by mapping countrywide screening data.

#### Continuum of care through supplementary services and intersectoral linkages

Apart from medical challenges, PLwRDs face host of challenges in school, jobs, and accessing public facilities in day-to-day life. Thus, RD management needs multi-sectoral involvement and holistic approach. RBSK envisions providing a continuum of care through intersectoral linkages and coordination, where various sectors such as education, social justice and empowerment, have an equal role to play in contributing to the child’s health and development. Different ministries such as the Ministry of Human Resource Development and the Ministry of Social Justice are also involved with DEICs to provide social services to the patients identified with 4Ds and their families. Under RBSK, DEICs are connected with different centers such as tertiary care centers for surgical needs, District Disability Rehabilitation Centre for people living with disabilities, and Block Resource Centers under Sarva Shiksha Abhiyan, a Government of India initiative for the universalization of elementary education, for the continuation of education.

#### Diagnostics, treatment, and management through referral services

DEIC aims to provide facilities for confirmatory diagnostics tests and early management through interdisciplinary early identification and intervention units and referrals to tertiary healthcare services. The medical team at DEIC usually consists of a pediatrician, dentist, audiologist, optometrist, speech therapist,, Auxiliary Nurse Midwifery, lab technicians, and psychotherapist. The referrals are dependent on the individual States/UTs mapping their healthcare resources. This resource directory is made available at DEICs.

The diagnostics odyssey of RDs take a long time. Moreover, facilities that provide RD treatment are far and few. The interdisciplinary approach to address a health condition under a single roof, access to a resource directory for the State/UT, and referral to the healthcare providers, if implemented efficiently, may prove to be extremely beneficial in reducing the time for PLwRDs to get diagnosed and have access to treatment. Once a child is identified to be showing certain anomalies, they can be referred to specialized centers like the Center of Excellence for RDS (CoEs) for specific diagnosis and treatment.

### Recommendations

The above discussion makes it clear that RBSK, although designed for common neonatal and childhood conditions in India, is intrinsically useful for RDs too. However, the scope of RBSK needs to be expanded further to include RDs as defined by NPRD. Here, we summarize a few recommendations that may help in integrating RD management more efficiently in RBSK.

1. **Expanding the capacity of RBSK**: At present RBSK covers thirty diseases (Supp1). However, if the scope of RBSK is expanded, the routine screening regime will help to identify any anomaly that signs the possibility of an RD. These children can be referred to tertiary care facilities and COEs for specific diagnoses.
2. **Enabling referral mechanism**: RBSK can play a significant role in screening children to identify possible cases of RDs. However specific diagnosis and treatment might not be possible through this program. Therefore, a strategic referral mechanism needs to be enacted to connect DEICs to the network of CoEs and National Inherited Disorders Administration(NIDAN) Kendras (translates to NIDAN centres) to achieve timely diagnosis and early intervention. Department of Biotechnology established the NIDAN Kendras to provide clinical care, focus towards providing prenatal testing, new-born screening, and genetic counselling for genetic disorders [16]. There should be a provision for teleconsultation within RBSK, especially in the cases in which tertiary healthcare is located far. This will be particularly helpful for PLwRDs who need to be provided a lifelong continuum of care [17]. In such cases, local healthcare providers can get virtual assistance from CoEs.
3. **Expanding target group to cover every child in the country through public-private partnership**: Present target group is limited to only children from the socioeconomic backward population and rural areas. However, the diagnostic odyssey of RDs is common among urban population and economically affluent classes as well, because of the absence of comprehensive screening programs. Further, the latest National sample survey found 61% of urban population largely accesses private healthcare systems [18]. Thus implementing RBSK guidelines through private healthcare facilities will enable larger access and reduce the burden on public healthcare systems which can focus on economically weaker sections.
4. **Enabling resource mapping**: Existing resources for RD management are scarce, dispersed across the country and not mapped in a common platform [7]. The resources mapping for RDs tertiary healthcare should be undertaken at the national level and private partners could be involved in select areas with special provisions. For many RDs, it is quite possible that the expertise to manage such cases may be available only at select centers in the country. Provisions should be made for easy referral to these centers.
5. **Generating Awareness**: There is a huge lack of awareness about RDs among general public, medical fraternity, policy makers and other relevant stakeholders [7, 19]. Awareness and capacity building on RDs are required among healthcare workers and professionals in India. Not only medical professionals but awareness and training about RDs can be extended to the entire RBSK network, for example, Ayurveda, Yoga and Naturopathy, Unani, Siddha and Homeopathy (AYUSH ) doctors, Mobile Health Team, and ASHA.
6. **Expanding services for genetic counseling**: RBSK advocates counseling for genetic disorders. However, a clear framework of how genetic tests need to be done, how the RBSK team has to be trained on genetic disorders, etc. has not been laid out. For making genetic services available and accessible it would be useful to connect the RBSK system and DEICs with the NIDAN Kendras.
7. **Expanding genetic diagnostic services**: The genetic diagnostic services capacity needs to be increased for the proper management of RDs. NPRD 2021 has also focused on this and directed capacity building in genetic services. Taking this forward, private genetic diagnostics services could be incorporated via MoU with DEICs, especially in places where genetic services capacity is limited in the public health settings. However, in doing so the cost of sequencing technology with private partners need to be regulated so that it is accessible to most patients.
8. **Enabling comprehensive data repository**: Importance of registry for RDs has been well acknowledged globally [20]. RBSK can enable the inclusion of comprehensive family history data prior to diagnosis. Family history data aside, more comprehensive data need to be recorded, that include maternal age and health (for neonates), spacing between birth, medications to mother during pregnancy and lactation, immunization, etc. A proper framework to integrate genetic diagnostic data within the larger data repository also needs to be figured out. This doubles up as public health surveillance data for teratogenic or infectious causes of common diseases and in some cases, RDs. RBSK needs to focus on seamless data management strategies while ensuring the principles of the National Digital Health Mission which recommends interoperability between independent and decentralized information systems while enhancing the security and privacy of the personal data of individuals. RBSK can form a crucial input system for the National Rare disease registry.
9. **Followup of SNCU graduates**: According to an estimate, 30% of children discharged from Special Newborn Care Units (SNCU) suffer from a lifelong disability and/or rare condition [12]. These children need to be routinely followed for developmental goals and other vital check-ups. Under the Home-Based Care for Young Children program, there is a provision for ASHA workers to visit and keep a record of such children till 15 months from their birth. However, for children with RDs, or who were admitted to Special Newborn Care Units and have a family history of RDs, this period may be extended till 6 years of age, till these children are admitted to schools and inducted into the RBSK system.
10. **Integrating patient groups in healthcare delivery**: As RDs are not well-understood, the experiences of PLwRDs provide valuable information for patients, caregivers, and medical professionals [7]. Globally patient groups have played an important role in shaping the healthcare system for RDs [21, 22]. Systematic approaches for integrating patient groups in public healthcare system has been studied and implemented in many countries [20]. RBSK can learn from these experiences and integrate patient groups formally into the healthcare delivery system by connecting diagnosed patients and clinicians to these groups.

## Conclusion

From the above discussion, it is evident that RBSK can rightly address the diagnostic odyssey faced by most PLwRDs through its early and comprehensive screening strategies. If RBSK is reinforced in the light of addressing a wider spectrum of diseases including RDs and implemented across the country it can prove to be a strong mechanism for prevention and limiting the effect of many childhood disorders. In India, health is a state subject and the implementation of a national program relies on state authorities and priorities. There has been a wide variation in the implementation of RBSK across different states [23]. However, in the interest of enhancing child health outcomes in the country, there should be a renewed momentum in rethinking the program and driving its implementation across states. An implementation framework needs to be established based on the learnings from states where RBSK has been implemented. Success of RBSK in addressing RDs in a vast country like India can serve as a role model for other countries with resource constrained healthcare system where specialized programs for RD management are not possible due to other competeing healthcare priorities. This study highlights features of RBSK that can efficiently manage RD which could be adapted in other resource constrained countries. Furthermore, this study can inspire identifcation and expansion of existing public healthcare programs for RD management in these low- and middle-income countries (LMICs) aswell.

## Limitations of the study

This study is based on the review of different RBSK primary resources made available by the government, secondary studies and interaction with key informants. Some aspects which are beyond the scope of this study and hence have not been considered are:

i) Challenges faced during the implementation of RBSK.
ii) Variations of the program as adopted in different Indian states.

## Electronic supplementary material

Below is the link to the electronic supplementary material.

Supplementary Material 1

## Data Availability

Data sharing not applicable to this article as no datasets were generated or analysed during the current study.

## Abbreviations

RMNCH + A: Reproductive, Maternal, Newborn, Child, and Adolescent Health of National Health Mission
RBSK: Rashtriya Bal Suraksha Karyakram
DEIC: District Early Intervention Centers
ASHA: Accredited Social Health Activist
PLwRDs: People living with RDs
NPRD: National Policy for Rare Diseases
CoEs: Center of Excellence for RDS
NIDAN: National Inherited Disorders Administration Kendras
SNCU: Special Newborn Care Units
AYUSH: Ayurveda, Yoga and Naturopathy, Unani, Siddha and Homeopathy

## References

[1] Nguengang Wakap S, Lambert DM, Olry A, Rodwell C, Gueydan C, Lanneau V, et al. Estimating cumulative point prevalence of rare diseases: analysis of the Orphanet database. Eur J Hum Genet. 2020 Feb;28(2):165–73. 10.1038/s41431-019-0508-0 PMC6974615 31527858

[2] Ministry of Health & Family Welfare. National Policy for Rare Diseases 2021 [Internet]. Government of India. ; 2021 Mar. Available from: https://main.mohfw.gov.in/sites/default/files/Final%20NPRD%2 C%202021.pdf.

[3] Bavisetty S, Grody WW, Yazdani S. Emergence of pediatric rare diseases: review of present policies and opportunities for improvement. Rare Dis. 2013 Jan;1(1):e23579. 10.4161/rdis.23579 PMC3932940 25002987

[4] EUROCAT. Congenital Anomalies are a Major Group of Mainly Rare Diseases [Internet]. 2012 [cited 2022 Apr 21]. Available from: https://eu-rd-platform.jrc.ec.europa.eu/sites/default/files/eurocat-pub-docs/Special-Report-Major-Group-of-Mainly-Rare-Diseases.pdf.

[5] UNICEF. Newborn and child health: Let’s end preventable neonatal deaths and ensure Every Child ALIVE. [Internet]. [cited 2021 Sep 2]. Available from: https://www.unicef.org/india/what-we-do/newborn-and-child-health.

[6] Infant Mortality Rate by Country. In Macrotrends LLC; [cited 2023 May 9]. Available from: https://www.macrotrends.net/countries/ranking/infant-mortality-rate.

[7] Choudhury MC, Saberwal G. The role of patient organizations in the rare disease ecosystem in India: an interview based study. Orphanet J Rare Dis. 2019 Dec;14(1):117. 10.1186/s13023-019-1093-6 PMC6542017 31142331

[8] Joszt L, Report. Economic Burden of Rare Diseases Is 10 Times Higher Than Mass Market Diseases [Internet]. AJMC. 2022 [cited 2022 Apr 25]. Available from: https://www.ajmc.com/view/report-economic-burden-of-rare-diseases-is-10-times-higher-than-mass-market-diseases.

[9] Choudhury MC, Chaube P. Integrating rare disease management in public health programs in India: exploring the potential of National Health Mission. Orphanet J Rare Dis. 2022 Dec;17(1):43. 10.1186/s13023-022-02194-z PMC8832777 35144652

[10] Operational Guidelines: Rashtriya Bal Swasthya Karyakram (RBSK). : Child Health Screening and Early Intervention Services under NRHM [Internet]. National Rural Health Mission; [cited 2021 Sep 2]. Available from: https://nhm.gov.in/images/pdf/programmes/RBSK/Operational_Guidelines/Operational%20Guidelines_RBSK.pdf.

[11] Wright CF, FitzPatrick DR, Firth HV. Paediatric genomics: diagnosing rare disease in children. Nat Rev Genet. 2018 May;19(5):253–68. 10.1038/nrg.2017.116 29398702

[12] Singh AK, Kumar R, Mishra CK, Khera A, Srivastava A. Moving from Survival to Healthy Survival through Child Health Screening and early intervention services under Rashtriya Bal Swasthya Karyakram (RBSK). Indian J Pediatr. 2015 Nov;82(11):1012–8. 10.1007/s12098-015-1823-2 26199076

[13] Government of Goa. The Goa Newborn Screening Program [Internet]. 2011 Sep [cited 2021 Sep 5]. Available from: http://www.dhsgoa.gov.in/documents/new_born.pdf.

[14] Bagcchi S. India has low doctor to patient ratio, study finds. BMJ. 2015 Sep;30:351:h5195. 10.1136/bmj.h5195 26423832

[15] National Family Health. Survey 2019–2021 [Internet]. Ministry of Health and Family Welfare, Government of India; 2021 [cited 2023 May 5]. Available from: https://main.mohfw.gov.in/sites/default/files/NFHS-5_Phase-II_0.pdf.

[16] Department of Biotechnology. Unique Methods of Management and treatment of Inherited Disorders (UMMID). An initiative of the Department of Biotechnology [Internet]. Ministry of Science & Technology, Government of India. ; 2019. Available from: https://static.pib.gov.in/WriteReadData/userfiles/Final%20-UMMID%20Booklet.pdf.

[17] Scherr JF, Albright C, de los Reyes E. Utilizing telehealth to create a clinical model of care for patients with Batten disease and other rare diseases. Therapeutic Adv Rare Disease. 2021 Jan;2:263300402110385. 10.1177/26330040211038564 PMC10032454 37181116

[18] Ministry of Statistics and Programme Implementation. Executive Summary on Report- Health in India, NSS 75th round [Internet]. [cited 2022 May 21]. Available from: http://164.100.161.63/sites/default/files/announcements/Summary%20Analysis_Report_586_Health.pdf?download=1.

[19] Agrawal R, Amaresh Rao M, Brian M, Chowdary G, Gayatri K, Krishnaji Rao M et al. Baseline Knowledge of Rare Diseases in India - A Survey. Int J Rare Dis Disord [Internet]. 2019 Aug 29 [cited 2022 May 23];2(1). Available from: https://www.clinmedjournals.org/articles/ijrdd/international-journal-of-rare-diseases-and-disorders-ijrdd-2-008.php?jid=ijrdd.

[20] Forsythe LP, Szydlowski V, Murad MH, Ip S, Wang Z, Elraiyah TA, et al. A systematic review of approaches for engaging patients for Research on Rare Diseases. J GEN INTERN MED. 2014 Aug;29(S3):788–800. 10.1007/s11606-014-2895-9 PMC4124116 25047393

[21] Black AP, Baker M. The impact of parent advocacy groups, the Internet, and social networking on rare diseases: The IDEA League and IDEA League United Kingdom example: The Impact of Parent Advocacy Groups. Epilepsia. 2011 Apr;52:102–4. 10.1111/j.1528-1167.2011.03013.x 21463291

[22] for the Rare Diseases Clinical Research Network, Merkel PA, Manion M, Gopal-Srivastava R, Groft S, Jinnah HA, et al. The partnership of patient advocacy groups and clinical investigators in the rare diseases clinical research network. Orphanet J Rare Dis. 2016 Dec;11(1):66. 10.1186/s13023-016-0445-8 PMC4870759 27194034

[23] Deloitte UNICEF. Formative Research Report on RBSK ‘From Survival to Healthy Survival’ [Internet]. 2016. Available from: http://nhm.gov.in/images/pdf/programmes/RBSK/RBSK_IEC/Formative_Research_Report_on_RBSK.pdf.

