# Supplementary material for: Expansion of India’s national child healthcare programme, Rashtriya Bal Swasthya Karyakram (RBSK), for rare disease management : a health policy perspective
Source: Orphanet J Rare Dis. 2023 Jun 12;18:145. doi: 10.1186/s13023-023-02761-y (PMC10262469; doi:10.1186/s13023-023-02761-y)
Supplement: Supplementary file 1 — Supplementary Material 1 [file 13023_2023_2761_MOESM1_ESM.docx]

**Brief description of working of RBSK**

At the birth facility, newborns are subjected to physical screening by a nurse/medical professional. The comprehensive physical screening is undertaken as per the RBSK handbook which includes recording vital signs, recording anthropometric data, checking for physical deformities, and responding to stimuli.  Under the Home Birth Newborn Care program, from the ages 0 to six weeks, ASHA visits the homes of the newborns and records their health data as per the RBSK handbook. ASHA can refer the children to DEICs in case they find a child has an underlying health condition. From the ages of 6 weeks to 6 years, children are screened twice a year at Anganwadi centers through physical examination and other non-invasive methods for disabilities, deficiencies, and developmental delays. From the ages of six to eighteen years, children are screened once a year at schools by mobile health teams (MHT) for 4Ds that also include parameters such as signs of depression and school performance. MHT consists of a female and a male AYUSH doctor, and ANM/pharmacist. For the child health screening, MHT is provided with transport and the necessary equipment to aid screening processes. After screening, the identified patients are referred to DEICs for further diagnosis and treatment. At DEICs, patients may be further referred to secondary and tertiary healthcare facilities. The cost of screening, diagnosis, treatment, and transport to and for from DEICs are covered under RBSK.
